# 97 Policy Framework for Integrating Sports Technology in Taiwan’s All-Age Environments and Supportive Elderly Exercise Spaces

**DOI:** 10.1093/eurpub/ckae114.022

**Published:** 2024-09-26

**Authors:** Chao-Chun Wu, Shu-Li Chia, Chia-Hui Lee, Chen-Su Lin, Shiang-Yun Huang, Shu-Li Chia, Tsung-Lin Li, Shiao-Chi Wu, Yu-Wen Huang

**Affiliations:** Health Promotion Administration, Ministry Of Health And Welfare, Taipei, Taiwan; Health Promotion Administration, Ministry Of Health And Welfare, Taipei, Taiwan; Health Promotion Administration, Ministry Of Health And Welfare, Taipei, Taiwan; Health Promotion Administration, Ministry Of Health And Welfare, Taipei, Taiwan; Health Promotion Administration, Ministry Of Health And Welfare, Taipei, Taiwan; Health Promotion Administration, Ministry Of Health And Welfare, Taipei, Taiwan; Health Promotion Administration, Ministry Of Health And Welfare, Taipei, Taiwan; Health Promotion Administration, Ministry Of Health And Welfare, Taipei, Taiwan; Health Promotion Administration, Ministry Of Health And Welfare, Taipei, Taiwan

## Abstract

**Purpose:**

Over 52.7% of Taiwan’s population aged 18 and above meet the WHO’s physical activity standards. This project aims to utilize technology-assisted recommendations to bridge the gap in sports-related resources among different regions, thereby boosting overall sports physical activity rates. Moreover, with Taiwan having entered an aged society in 2018, the initiative involves promoting fitness clubs for elders within communities to enhance the well-being of elderly individuals dealing with health issues, sub-health conditions, or frailty. These clubs aim to improve participants’ muscle strength, cognitive function, and social engagement through increased physical activity.

**Project or policy description:**

Since 2020, Taiwan has been implementing the silver fitness club program where local administrations strategically utilize idle public spaces in accordance with local conditions. These clubs receive support from sports instructors who deliver diverse health promotion courses, aiming to increase the accessibility of physical activities for elders in communities as well as ensure balanced development of physical activity resources between urban and rural areas.

Moreover, since 2021, Taiwan has been integrating emerging sports technology (such as motion detection, exercise data recording apps) into different communities and medical institutional settings. Through automatic API uploading to data platform, we analyze physiological and exercise data, integrating health behaviors, statuses, services, and environments. By aligning exercise with wellness, targeted exercise training programs with health topics are offered. Intelligent health not only enhances service efficiency but also demonstrates well cross-departmental collaboration (between the Ministry of Health and Welfare and the Ministry of Digital Affairs) and vertical integration (between central and local governments).

**Conclusions:**

As of 2023, Taiwan has set up 16 sports technology hubs and 128 Silver fitness clubs across all 21 counties and cities, aiming to widen access to sports technology experiences and reduce barriers to participation. Approximately 150,000 visits have made with data analysis providing tailored exercise recommendations. Presently, over 90% of elder participants in these fitness clubs report noticeable positive enhancements in mental and muscular strength, meeting the objectives of disability prevention and delay.

This work was funded by the Health Promotion Administration, Ministry of Health and Welfare. Program number MP11202-0098.

